# Kinetic and Sequence-Structure-Function Analysis of Known LinA Variants with Different Hexachlorocyclohexane Isomers

**DOI:** 10.1371/journal.pone.0025128

**Published:** 2011-09-16

**Authors:** Pooja Sharma, Rinku Pandey, Kirti Kumari, Gunjan Pandey, Colin J. Jackson, Robyn J. Russell, John G. Oakeshott, Rup Lal

**Affiliations:** 1 Department of Zoology, University of Delhi, Delhi, India; 2 CSIRO Ecosystem Sciences, Canberra, Australia; 3 Research School of Chemistry, Australian National University, Canberra, Australia; Charité-University Medicine Berlin, Germany

## Abstract

**Background:**

Here we report specific activities of all seven naturally occurring LinA variants towards three different isomers, α, γ and δ, of a priority persistent pollutant, hexachlorocyclohexane (HCH). Sequence-structure-function differences contributing to the differences in their stereospecificity for α-, γ-, and δ-HCH and enantiospecificity for (+)- and (−)-α -HCH are also discussed.

**Methodology/Principal Findings:**

Enzyme kinetic studies were performed with purified LinA variants. Models of LinA2_B90A_ A110T, A111C, A110T/A111C and LinA1_B90A_ were constructed using the FoldX computer algorithm. Turnover rates (min^−1^) showed that the LinAs exhibited differential substrate affinity amongst the four HCH isomers tested. α-HCH was found to be the most preferred substrate by all LinA's, followed by the γ and then δ isomer.

**Conclusions/Significance:**

The kinetic observations suggest that LinA-γ1-7 is the best variant for developing an enzyme-based bioremediation technology for HCH. The majority of the sequence variation in the various *linA* genes that have been isolated is not neutral, but alters the enantio- and stereoselectivity of the encoded proteins.

## Introduction

Hexachlorocyclohexane (HCH) consists of four main isomers (α-, β-, γ- and δ-HCH), all of which are highly toxic to vertebrates and one of which (γ-HCH; lindane) is a potent insecticide [1]. Toxicity concerns have led to deregistration of lindane in many countries, and large dumps of unused HCH now pose major environmental hazards [2], [3], [4]. Bacterial strains have evolved to degrade HCH, with the initial steps in their degradation of the α-, γ- and δ- isomers catalyzed by the enzyme LinA [2]. The crystal structure of LinA shows that it shares a structural fold and an Asp-His catalytic dyad with enzymes of the scytalone dehydratase family [5], [6]. LinA catalyses E2 elimination reactions at biaxial H-Cl pairs of atoms in α-, γ- and δ- HCH [2], [7], [8]. Seven naturally occurring LinA variants have been identified (Table 1), which differ in as many as 10% of their residues. However, little is known of their functional differences other than that LinA1_B90A_ and LinA2_B90A_ preferentially catalyze degradation of the (+)-α-HCH and (−)-α-HCH enantiomers, respectively [2], [9]. Here we report the specific activities of all seven previously cloned *linA* gene variants towards α-, γ- and δ-HCH and analyse how their sequence differences contribute to differences in their activities.

**Table 1:** Turnover rates (min^−1^) of the published LinA variants towards α-, γ- and δ-HCH, along with the amino acid differences amongst them^a^.

			Residue variations																							Turnover number (min^−1^)			
			20	23	35	68	71	96	110	111	113	115	126	129	131	133	145	149	150	151	152	153	154	155	156	α	γ	α/γ	δ
**Group 1**	**A**	**LinA2_B90A_**	K	A	I	F	C	L	A	A	F	D	F	R	A	T	A	I	H	F	A	P	S	G	A	3815±3	733±13	5.2	174±4
		**LinA_γ1-7_**	**.**	**.**	**.**	**.**	**.**	**.**	T	C	**.**	**.**	**.**	**.**	**.**	**.**	**.**	**.**	**.**	**.**	**.**	**.**	**.**	**.**	**.**	3001±30	11794±526	0.2	284±32
		**LinAb_ITRC-5_**	**.**	**.**	**.**	**.**	**.**	**.**	T	C	**.**	**.**	**.**	**.**	**.**	**.**	**.**	**.**	**.**	**.**	T	**.**	**.**	**.**	**.**	2306±29	2778±180	0.8	246±3
		**LinA_NM05_**	**.**	**.**	**.**	**.**	**.**	**.**	T	C	**.**	**.**	**.**	**.**	**.**	**.**	**.**	**.**	**.**	L	**.**	**.**	T	**.**	**.**	11267±27	889±107	12.6	10±1
	**b**	**LinA_DS3-1_**	**.**	**.**	**.**	**.**	**.**	**.**	**.**	**.**	**.**	**.**	**.**	**.**	**.**	**.**	**.**	A	L	L	Q	K	**.**	**.**	**.**	7884±14	5376±359	1.4	214±35
**Group 2**	**a**	**LinAa_ITRC-5_**	**.**	G	V	Y	T	C	**.**	**.**	Y	N	**.**	L	G	M	V	A	**.**	**.**	**.**	**.**	**.**	–	–	965±0	65±15	14.8	49±1
	**b**	**LinA1_B90A_**	Q	G	**.**	Y	T	C	**.**	**.**	Y	N	L	L	G	M	**.**	A	L	L	Q	K	**.**	–	–	418±4	89±7	4.6	10±1

a LinA2_B90A_ derives from *Sphingobium indicum* B90A and is identical to LinA from *Sphinogobium* sps. strains UT26, Sp+, DS2, γ16-1, BHC-A and *Rhodanobacter lindaniclasticus*; LinA_γ1-7_ is from *Sphingomona*s sp. γ1-7; LinAb_ITRC-5_ is from *Pseudomonas aeruginosa* ITRC-5; LinA_NM05_ comes from *Sphingomonas* sp. NM05; LinA_DS3-1_ is from *Sphingomonas* sp. DS3-1 and is identical to LinA of *Sphingobium* sp. α1-2; LinAa_ITRC-5_ is from *Pseudomonas aeruginosa* ITRC-5; LinA1_B90A_ is from *Sphingobium indicum* B90A and is identical to LinA of *Sphingomonas* sp. α4-2 [2].

## Materials and Methods

Codon optimized *linA* genes for expression of all the variants in *E. coli* were synthesized by Geneart AG, Regensburg Germany (GenBank accession numbers HM447244–HM447250). The synthetic *linA* genes were PCR amplified with attB1, attB2 and attB2-R2 primers (Table S1) and the amplicons were then cloned into pDONR201 and transferred to pDEST17 using the BP and LR reactions, respectively, following the manufacturers' instructions (Invitrogen, CA). The host *E. coli* BL21-AI™ (Invitrogen) cells co-expressed chaperones from the plasmid pGro7 (Takara, Japan).

The bacterial clones were cultured in 100 ml of LB at 28°C [10]. When the culture reached an OD_600_ of 0.5, L-(+)-arabinose was added to a final concentration of 2 g/L. Cells were harvested after overnight incubation, washed with 10 mM imidazole buffer (pH 7.5) and disrupted by 1× bugbuster (Novagen, Darmstadt). The lysate was centrifuged at 16,000 g for 20 min and the supernatant was used to purify his-tagged proteins using NTA-Ni^2+^ agarose (Qiagen, GmbH) following the manufacturers' instructions. The purified protein was quantified using Nanodrop (Thermo Scientific, DE). The purified enzyme was stored in storage buffer (pH 7.5) containing 1 mM 2-mercaptoethanol and 10% glycerol at a concentration of about 1 mg/ml at 4°C.

Enzyme assays were performed within 3 days of purification. In this period no measurable loss of enzyme activity was observed (data not shown). LinA activity was assayed by estimating the depletion of HCH isomers using gas chromatography. The assay reaction was initiated by the addition of enzyme to a reaction mixture (500 µl) containing 1.7 µM of the respective HCH isomer in 1× Tris glycine buffer (25 mM Tris, 192 mM glycine pH 8.3) at 22°C and was stopped by the addition of 0.3% (v/v) formic acid (final concentration). The incubation times for reaction mixtures containing α- and γ-HCH as substrates were 30 sec and 1 min, respectively, and those for δ-HCH assays were 2 or 5 min, depending on the activity of the enzyme. The samples were extracted in an equal volume of hexane by vortexing for 5 min and quantitatively analyzed on a GC equipped with a BPX-50 capillary column (30 m by 0.25 mm by 0.32 µm; SGE Analytical) and an electron capture detector. The temperature program was isothermal at 100°C for 5 min, followed by an increase to 200°C at 20°C/min, and the carrier gas (He) flow rate was 3.0 ml/min.

Models of LinA2_B90A_ A110T, A111C, A110T/A111C and LinA1_B90A_ were constructed using the FoldX computer algorithm (http://foldx.crg.es/), which permits the construction of low-energy models and calculation of interaction energies contributing to the stability of proteins [11], [12]. These mutations were modeled using the available crystal structure of LinA2_B90A_ (PDB ID: 3A76). Prior to mutagenesis, the RepairPDB option of FoldX was used to optimize the total energy of the protein, which involved identifying and repairing residues with bad torsion angles and van der Waals clashes. Mutagenesis was subsequently performed using the BuildModel option of FoldX. The effects of the mutations on protein stability were obtained from the output file. The geometries of HCH isomers used in the docking analyses were taken from Brittain *et al.* [7]. The substrates were docked by superimposing them on the conformations of the transition states as docked in that work and with reference to the nearby histidine.

## Results and Discussion

Histidine-tagged LinA proteins were heterologously expressed in *E. coli* co-expressing the chaperone GroEL and purified to homogeneity by affinity chromatography as described by Brittain *et al.* [7]. Specific activities of the various proteins with α-, γ- and δ-HCH were measured using gas chromatography with electron capture detection. The seven variants fall into two main groups according to sequence differences at positions 20, 23, 35, 68, 71, 96, 113, 115, 126, 129, 131 and 133 and the first and larger group has higher activities for all isomers than those of the second group (Table 1). However there is also functionally important variation in two sections of the sequence within these groups: 110 and 111, where A–T and A–C differences co-occur, and at the C-terminus (149–156), where most sequences are either I-H-F-A-P-S-G-A or A-L-L-Q-K-S, or minor variants thereof. Several specific comparisons show the effects of these variable regions on isomer specific activities. Firstly, LinA2_B90A_ and LinA_γ1-7_, which differ only in the A110T/A111C pair, show radically different α-HCH:γ-HCH isomer specificities (5.2 *cf.* 0.25). Secondly, differences in the 149–154 region between LinA_γ1-7_, LinAb_ITRC-5_ and LinA_NM05_ also have a large effect on this ratio (0.25 *cf.* 14.8 and 12.6, respectively). Thirdly, differences in the 149–156 region between LinA2_B90A_ and LinA_DS3-1_ also have large effects on activity, with LinA_DS3-1_ being more active for all isomers, but particularly for γ-HCH (7 fold, *cf.* 2 fold for α-HCH). Interestingly, this C-terminal variation is thought to result from insertion of the IS*6100* transposable element [2], [13].

**Figure 1:**
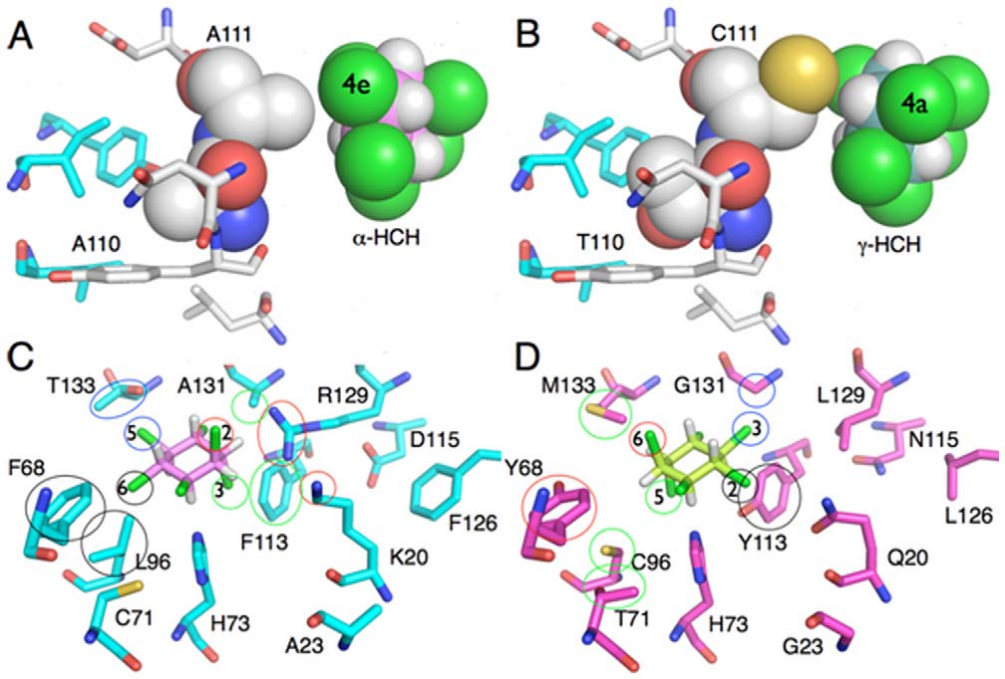
Effect of mutation and sequence differences in the LinA enzymes. The effect of the A111C mutation is shown in panels A and B, illustrating that the cysteine residue will clash with the equatorial chlorine at the 4-position of (+)-α-HCH (4e), but provides complementary contacts with γ-HCH when the chlorine at the 4-position is axial (4a). Panels C and D show the effects of the sequence differences between LinA2_B90A_ and LinA1_B90A_ on enantioselectivity, with (+)- and (−)-α-HCH docked in each active site. The important sequence differences in the LinA enzymes and structural differences in the α-HCH enantiomers are circled.

To investigate how the 110/111 sequence differences change the isomer specificity, the FoldX force-field [12] was used to create models of LinA_γ1-7_ and LinA1_B90A_ from the crystal structure of LinA2_B90A_ (PDB ID: 3A76) [5] and to estimate the stabilizing/destabilizing effects of these mutations (similar analysis could not be carried out for the C-terminal differences because the structure of that region is not well resolved). Fig. 1 shows that the reversal in α-HCH: γ-HCH isomer specificities (5.2 *cf.* 0.25) on changing from A110/A111 in LinA2_B90A_ to T110/C111 in LinA_γ1-7_ is a result of the A111C mutation, which is opposite the 3-position of the HCH ring, the only position where α- and γ-HCH differ. This mutation will provide closer, more favourable contact with γ-HCH but generate some level of steric clash with α-HCH. However, this A111C change is predicted to be highly destabilizing (ΔΔG 0.75 kcal/monomer), whereas A110T appears to provide a compensating, stabilizing effect (ΔΔG -1.28 kcal/monomer) by extending into a hydrophobic cavity at the trimer interface. These mutations thus provide a clear example of the role of stabilizing mutations (A110T) in allowing function-changing mutations (A111C), which would otherwise result in aggregation of the protein, to be tolerated [14].

All seven variants show the same broad isomer preferences as described previously for the LinA_UT26_ enzyme (identical sequence to LinA2_B90A_ above), i.e. high activity towards α- and γ-HCH and less activity towards δ-HCH [2], [8], [15], [16]. Quantitatively, however, there are large differences among the seven variants in their absolute and relative activities (Table 1): α-HCH turnover varies from 418 to 11267 min^−1^, γ-HCH turnover varies from 65 to 11795 min^−1^ and the ratio (α∶γ) varies from 14.8 to 0.25. Similarly -HCH activities show over 200 fold variation in absolute terms, with values ranging from ∼2 to 75% of the corresponding γ-HCH value in relative terms.

Suar *et al.* [9] have previously determined the enantioselectivity of certain LinA variants for the (+) and (−) enantiomers of α-HCH, finding LinA1_B90A_ and LinA2_B90Aδ_ have strong preferences for (+)- and (−)-α-HCH, respectively. LinA1_B90A_ and LinA2_B90A_ are typical of the two main sequence groups in Table 1 and we can now use the structure described by Okai *et al.* [5] to analyse how the 18 amino acid differences between these sequences may contribute to the enantioselectivity differences. To do this we docked the two enantiomers in the active site based on the transition state geometry of the elimination reaction [5]. The most noticeable difference between the structures is that the positive charge on R129, which is catalytically essential and provides stabilising interactions with the leaving group in the transition state for LinA2_B90A_ [5], is absent in LinA1_B90A_, with L129 unable to fulfil this role. The positively charged side chain of K20 is also found in this region in LinA2_B90A_ and may fulfil a similar, if less critical, role that also cannot be replicated by Q20 in LinA1_B90A_. These findings are consistent with the leaving group changing sides from the 2-position in LinA2 _B90A_ to the 6-position in LinA1_B90A_. Three other mutations in the second shell most likely compensate for these mutations (A23G, D115N, F126L). Other sequence differences account for changes in the position of the axial groups in the ring: F68Y provides a H-bonding group at the site of the axial leaving group in the 6-position, which will stabilize the transition state; the C71T/L96C/T133M mutations reshape the active site to account for the equatorial chlorine in the 5-position of (−)-α-HCH being axial in (+)-α-HCH; and similarly, the F113Y difference fills the space formed from the axial chlorine at the 3-position of (−)-α-HCH changing to equatorial in (+)-α-HCH. P153K might be involved in leaving group stabilization to fulfil an analogous role to that which K20 and R129 perform in LinA2 _B90A_.

Our analysis suggests that a majority of the sequence variation in the various *linA* genes that have been isolated is not neutral, but alters the enantio- and stereoselectivity of the encoded proteins. Significantly, some organisms, for example *Sphingobium indicum* B90A and *Pseudomonas aeruginosa* ITRC5, have at least two copies of the genes, in each case one from each of the two major sequence groups, thus providing those organisms with enhanced substrate ranges, covering α- and HCH as well as (+)-α- and (−)-α-HCH. Notably, *linA* has a different codon bias and G+C content than other *lin* genes encoding subsequent steps in the HCH degradation pathway [2], suggesting that it may be recently acquired. It will be interesting to monitor ongoing evolution of the *lin* system, and in particular LinA, to see whether this recently emerged pathway continues to adapt to the challenges of life in soils polluted with HCH.

## Supporting Information

Table S1 ***lin*** **A specific primers used in the current study to clone** ***lin*** **A variants into gateway vector pDONR201 (Invitrogen).**
(DOC)

## References

[1] Slade RE (1945). The gamma isomer of hexachlorocyclohexane (gammexane). An insecticide with outstanding properties.. Chem Ind.

[2] Lal R, Pandey G, Sharma P, Kumari K, Malhotra S (2010). Biochemistry of microbial degradation of hexachlorocyclohexane and prospects for bioremediation.. Microbiol Mol Biol Rev.

[3] Phillips TM, Lee H, Trevors JT, Seech AG (2006). Full-scale *in situ* bioremediation of hexachlorocyclohexane-contaminated soil.. J Chem Technol Biotechnol.

[4] Rubinos DA, Villasuso R, Muniategui S, Barral MT, Diaz Fierros F (2007). Using the landfarming technique to remediate soils contaminated with hexachlorocyclohexane isomers.. Water Air Soil Pollut.

[5] Okai M, Kubota K, Fukuda M, Nagata Y, Nagata K (2010). Crystal structure of γ-hexachlorocyclohexane dehydrochlorinase LinA from *Sphingobium japonicum* UT26.. J Mol Biol.

[6] Nagata Y, Mori K, Takagi M, Murzin AG, Damborsky J (2001). Identification of protein fold and catalytic residues of γ-hexachlorocyclohexane dehydrochlorinase LinA.. Proteins.

[7] Brittain DRB, Pandey R, Kumari K, Sharma P, Pandey G (2011). Competing S_N_2 and E2 reaction pathways for hexachlorocyclohexane degradation in the gas phase, solution and enzymes.. Chem Commun.

[8] Trantirek L, Hynkova K, Nagata Y, Murzin A, Ansorgova A (2001). Reaction mechanism and stereochemistry of γ-hexachlorocyclohexane dehydrochlorinase LinA.. J Biol Chem.

[9] Suar M, Hauser A, Poiger T, Buser HR, Muller MD (2005). Enantioselective transformation of alpha-hexachlorocyclohexane by the dehydrochlorinases LinA1 and LinA2 from the soil bacterium *Sphingomonas paucimobilis* B90A.. Appl Environ Microbiol.

[10] Sambrook J, Fritsch EF, Maniatis T (1989). Molecular Cloning: A Laboratory Manual.

[11] Guerois R, Nielsen JE, Serrano L (2002). Predicting changes in the stability of proteins and protein complexes: a study of more than 1000 mutations.. J Mol Biol.

[12] Schymkowitz J, Borg J, Stricher F, Nys R, Rousseau F (2005). The FoldX web server: an online force field.. Nucleic Acids Res.

[13] Lal R, Dogra C, Malhotra S, Sharma P, Pal R (2006). Diversity, distribution and divergence of *lin* genes in hexachlorocyclohexane-degrading sphingomonads.. Trends Biotechnol.

[14] Bershtein S, Segal M, Bekerman R, Tokuriki N, Tawfik DS (2006). Robustness-epistasis link shapes the fitness landscape of a randomly drifting protein.. Nature.

[15] Nagata Y, Miyauchi K, Takagi M (1999). Complete analysis of genes and enzymes for γ-hexachlorocyclohexane degradation in *Sphingomonas paucimobilis* UT26.. J Ind Microbiol Biotechnol.

[16] Nagata Y, Hatta T, Imai R, Kimbara K, Fukuda M (1993). Purification and characterization of γ-hexachlorocyclohexane (γ-HCH) dehydrochlorinase (LinA) from *Pseudomonas paucimobilis*.. Biosci Biotech Biochem.

