# Supplementary material for: Kinetic and Sequence-Structure-Function Analysis of Known LinA Variants with Different Hexachlorocyclohexane Isomers
Source: PLoS One. 2011 Sep 16;6(9):e25128. doi: 10.1371/journal.pone.0025128 (PMC3174995; doi:10.1371/journal.pone.0025128)
Supplement: Table S1 — lin A specific primers used in the current study to clone lin A variants into gateway vector pDONR201 (Invitrogen). (DOC) [file pone.0025128.s001.doc]

**Table S1. *lin*A specific primers used in the current study to clone *lin*A variants into gateway vector pDONR201 (Invitrogen)**

| **Primer** | **Sequence (5’ to 3’)** |  |
| --- | --- | --- |
| linA2-CO-attB1 | GGGGACAAGTTTGTACAAAAAAGCAGGCTTAATGAGCGATCTGGATCGT | Forward primer for all *lin*A’s |
| linA2-CO-attB2 | GGGGACCACTTTGTACAAGAAAGCTGGGTATCATTACGCGCCGCTCGG | Reverse primer for  *lin*A2_B90A_, *lin*A_γ1-7_,  *lin*A_NM05_, *lin*Aa _ITRC-5_ & *lin*Ab_ITRC-5_ |
| linA2-CO-attB2-R2 | GGGGACCACTTTGTACAAGAAAGCTGGGTATCATTAGCTCTTCTGCAGC | Reverse primer for *lin*A_DS3-1_ & *lin*A1_B90A_ |
